# Supplementary material for: Decreased Innate Migration of Pro-Inflammatory M1 Macrophages through the Mesothelial Membrane Is Affected by Ceramide Kinase and Ceramide 1-P
Source: Int J Mol Sci. 2022 Dec 15;23(24):15977. doi: 10.3390/ijms232415977 (PMC9785226; doi:10.3390/ijms232415977)
Supplement: Supplementary file 1 [file ijms-23-15977-s001.zip › ijms-2032672-supplementary.pdf]

Table S1: Summary of 45 measured cytokines, chemokines and growth factors' sensitivity, Lower and Upper Limit of Quantification (LLOQ, ULOQ).

*IL-4, IL-13 and IFNg were excluded from data analysis because they were added into culture for macrophage polarization.

Cytokine	Sensitivity	LLOQ	ULOQ	Detected?	Excluded from analysis*
BDNF	1.02 pg/mL	0.93pg/mL	10000 pg/mL	Yes	
EGF	0.85 pg/mL	2.08 pg/mL	12000 pg/mL	Yes	
Eotaxin	1.4 pg/mL	0.61 pg/mL	2500 pg/mL	Yes	
FGF-2	2.47 pg/mL	1.14 pg/mL	20000 pg/mL	Yes	
GM-CSF	1.2 pg/mL	17.09 pg/mL	70000 pg/mL	No	
GROα	2.8 pg/mL	2.44 pg/mL	10000 pg/mL	Yes	
HGF	3.10 pg/mL	8.59 pg/mL	36000 pg/mL	Yes	
IFNα	0.2 pg/mL	0.61 pg/mL	2500 pg/mL	Yes	
IFNγ	0.2 pg/mL	10.69 pg/mL	50000 pg/mL	Yes	Yes
IL-1RA	17.8 pg/mL	34.18 pg/mL	140000 pg/mL	Yes	
IL-1α	0.1 pg/mL	0.57 pg/mL	2500 pg/mL	Yes	
IL-1β	0.2 pg/mL	2.08 pg/mL	10000 pg/mL	Yes	
IL-2	0.8 pg/mL	4.48 pg/mL	20000 pg/mL	Yes	
IL-4	1.5 pg/mL	10.98 pg/mL	50000 pg/mL	No	Yes
IL-5	0.3 pg/mL	4.72 pg/mL	30000 pg/mL	Yes	
IL-6	0.4 pg/mL	9.6 pg/mL	40000 pg/mL	Yes	
IL-7	0.2 pg/mL	0.31 pg/mL	2500 pg/mL	Yes	
IL-8	1.2 pg/mL	2.44 pg/mL	10000 pg/mL	Yes	
IL-9	0.5 pg/mL	9.77 pg/mL	40000 pg/mL	Yes	
IL-10	0.1 pg/mL	2.29 pg/mL	10000 pg/mL	Yes	
IL-12p70	0.04 pg/mL	6.28 pg/mL	28000 pg/mL	Yes	
IL-13	0.1 pg/mL	2.44 pg/mL	10000 pg/mL	No	Yes
IL-15	1.1 pg/mL	2.06 pg/mL	12500 pg/mL	Yes	
IL-17A	0.1 pg/mL	1.69 pg/mL	10000 pg/mL	No	
IL-18	0.4 pg/mL	8.88 pg/mL	40000 pg/mL	Yes	
IL-21	0.6 pg/mL	9.61 pg/mL	40000 pg/mL	Yes	
IL-22	8.2 pg/mL	31.74 pg/mL	130000 pg/mL	Yes	
IL-23	0.9 pg/mL	14.59 pg/mL	60000 pg/mL	Yes	
IL-27	5.1 pg/mL	15.3 pg/mL	100000 pg/mL	Yes	
IL-31	3.3 pg/mL	14.17 pg/mL	80000 pg/mL	Yes	
IP-10	0.3 pg/mL	1.95 pg/mL	8000 pg/mL	Yes	
LIF	0.83 pg/mL	5.53 pg/mL	25000 pg/mL	Yes	
MCP-1	0.6 pg/mL	1.22 pg/mL	5000 pg/mL	Yes	
MIP-1α	1.1 pg/mL	2.14 pg/mL	8750 pg/mL	No	
MIP-1β	4.7 pg/mL	6.10 pg/mL	25000 pg/mL	No	
NGFβ	6.19 pg/mL	7.32 pg/mL	30000 pg/mL	Yes	
RANTES	0.2 pg/mL	0.61 pg/mL	2500 pg/mL	Yes	
PDGF-BB	6.01 pg/mL	1.94 pg/mL	30000 pg/mL	Yes	
PIGF-1	0.23 pg/mL	0.71 pg/mL	5000 pg/mL	Yes	
SCF	0.57 pg/mL	1.37 pg/mL	6500 pg/mL	Yes	
SDF-1α	20.5 pg/mL	17.09 pg/mL	70000 pg/mL	Yes	
TNFα	0.4 pg/mL	7.8 pg/mL	35000 pg/mL	Yes	
TNFβ	1.6 pg/mL	1.34 pg/mL	25000 pg/mL	Yes	
VEGF-A	1.73 pg/mL	5.86 pg/mL	24000 pg/mL	Yes	

VEGF-D	0.88 pg/mL	12.21 pg/mL	50000 pg/mL	Yes

Table S2: Fold change ratio of cytokines between macr

Cytokine	M1/M0	M2/M0	M2/M1
BDNF	1.31	1.36	1.04
EGF	0.84	0.81	0.97
Eotaxin	0.95	0.81	0.85
FGF-2	2.54	0.88	0.35
GRO-α	0.12	0.076	0.62
HGF	1.23	0.62	0.51
IFNα	1.24	1.2	0.96
IL-1RA	1.18	0.93	0.79
IL-1α	1.22	0.56	0.46
IL-1β	13.89	2.3	0.17
IL-2	1.56	0.91	0.58
IL-21	4.61	0.34	0.07
IL-22	2.35	0.51	0.22
IL-23	27.14	0.29	0.01
IL-27	1.34	0.78	0.58
IL-31	2.08	NA	NA
IL-5	1.72	4.91	2.86
IL-6	1.65	0.56	0.34
IL-7	2.74	0.32	0.12
IL-8	0.48	1.19	2.50
IL-9	0.82	0.41	0.50
IL-10	0.02	0.63	30.30
IL12p70	16.27	1.39	0.09
IL-15	1.9	0.53	0.28
IL-18	38.51	224.97	5.88
IP-10	1.37	0.66	0.48
LIF	4.28	14.09	3.33
MCP-1	0.85	0.82	0.96
NGFβ	0.96	1.24	1.30
PDGF-BB	10.31	17.68	1.72
PIGF-1	1.06	0.81	0.76
RANTES	0.64	1.12	1.75
SCF	1.36	1.72	1.27
SDF-1α	0.83	0.62	0.75
TNF-α	30.02	2.12	0.07
TNF-β	9.46	0.13	0.01
VEGF-A	0.86	1.29	1.52
VEGF-D	0.49	0.67	1.37

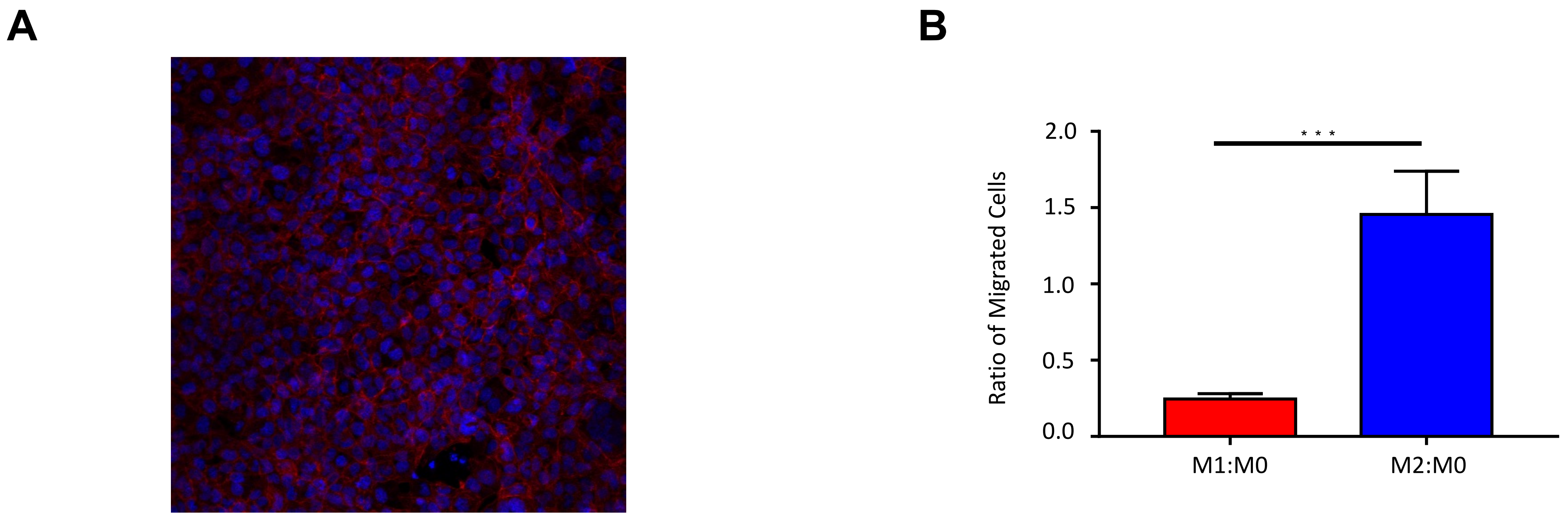

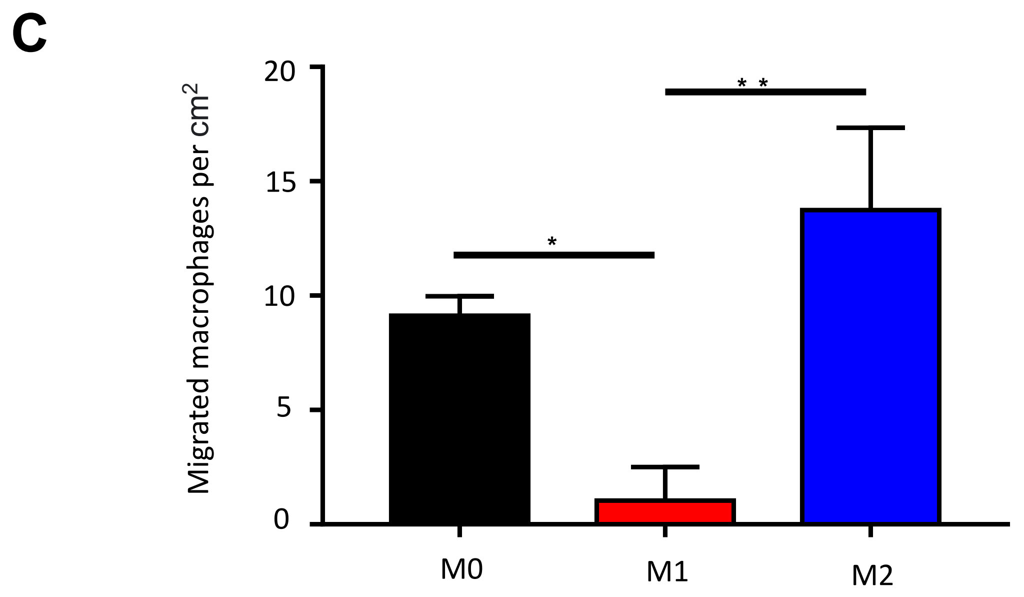

Figure S1. *, $p < 0.05$, **, $p < 0.01$, ***, $p < 0.005$

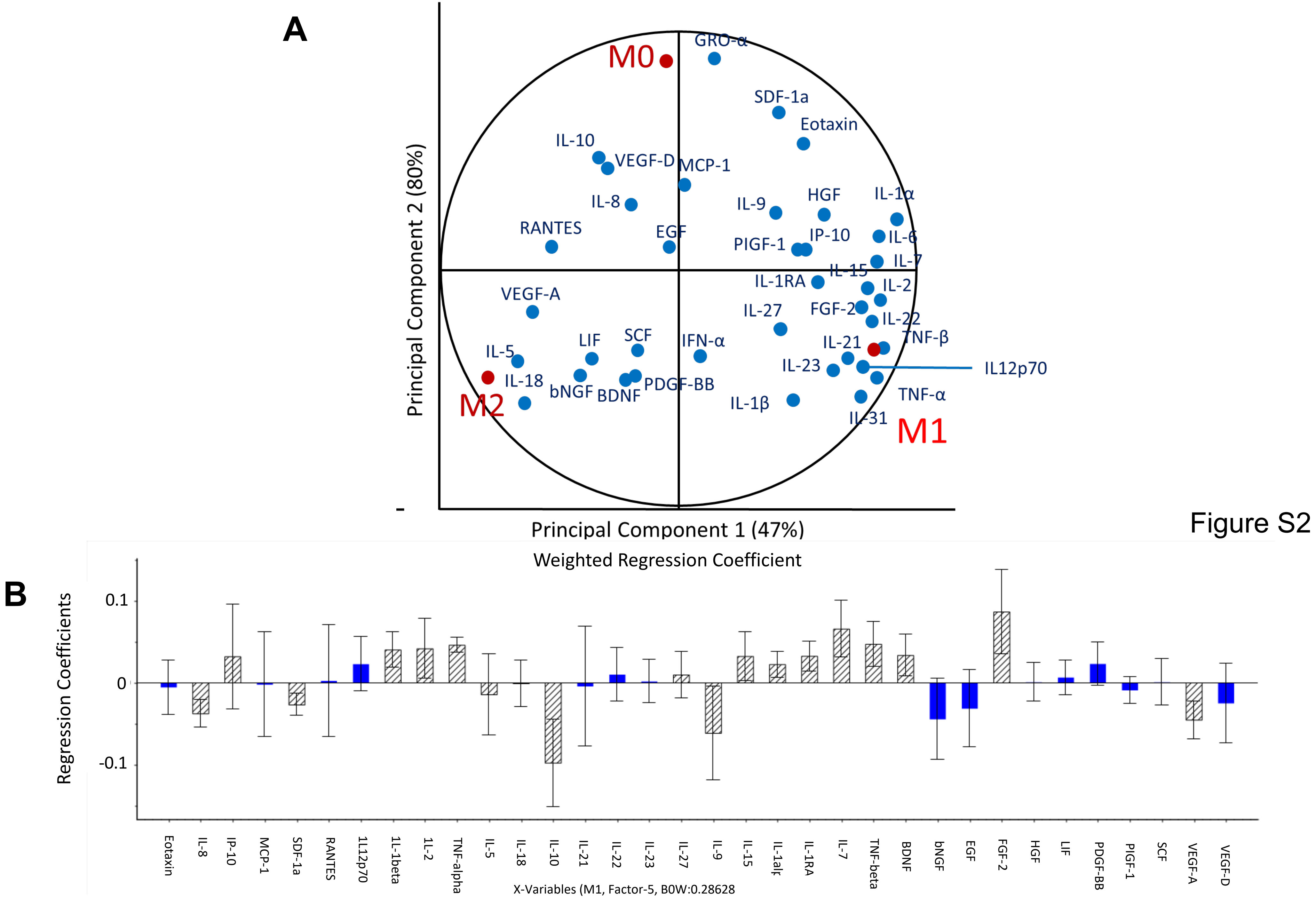

A
M0
M1
M2
GRO-α
SDF-1a
Eotaxin
IL-10
VEGF-D
MCP-1
IL-8
IL-9
HGF
IL-1α
RANTES
EGF
PIGF-1
IP-10
IL-6
IL-7
IL-15
IL-1RA
IL-2
VEGF-A
IL-27
FGF-2
IL-22
TNF-β
IL-21
IL-23
IL12p70
LIF
SCF
IFN-α
IL-5
IL-18
bNGF
BDNF
PDGF-BB
IL-1β
TNF-α
IL-31
Principal Component 2 (80%)
Principal Component 1 (47%)
B
Weighted Regression Coefficient
Regression Coefficients
0.1
0
-0.1
Eotaxin
IL-8
IP-10
MCP-1
SDF-1a
RANTES
1L12p70
1L-1beta
1L-2
TNF-alpha
IL-5
IL-18
IL-10
IL-21
IL-22
IL-23
IL-27
IL-9
IL-15
IL-1alk
IL-1RA
IL-7
TNF-beta
BDNF
bNGF
EGF
FGF-2
HGF
LIF
PDGF-BB
PIGF-1
SCF
VEGF-A
VEGF-D
X-Variables (M1, Factor-5, B0W:0.28628)

Figure S2

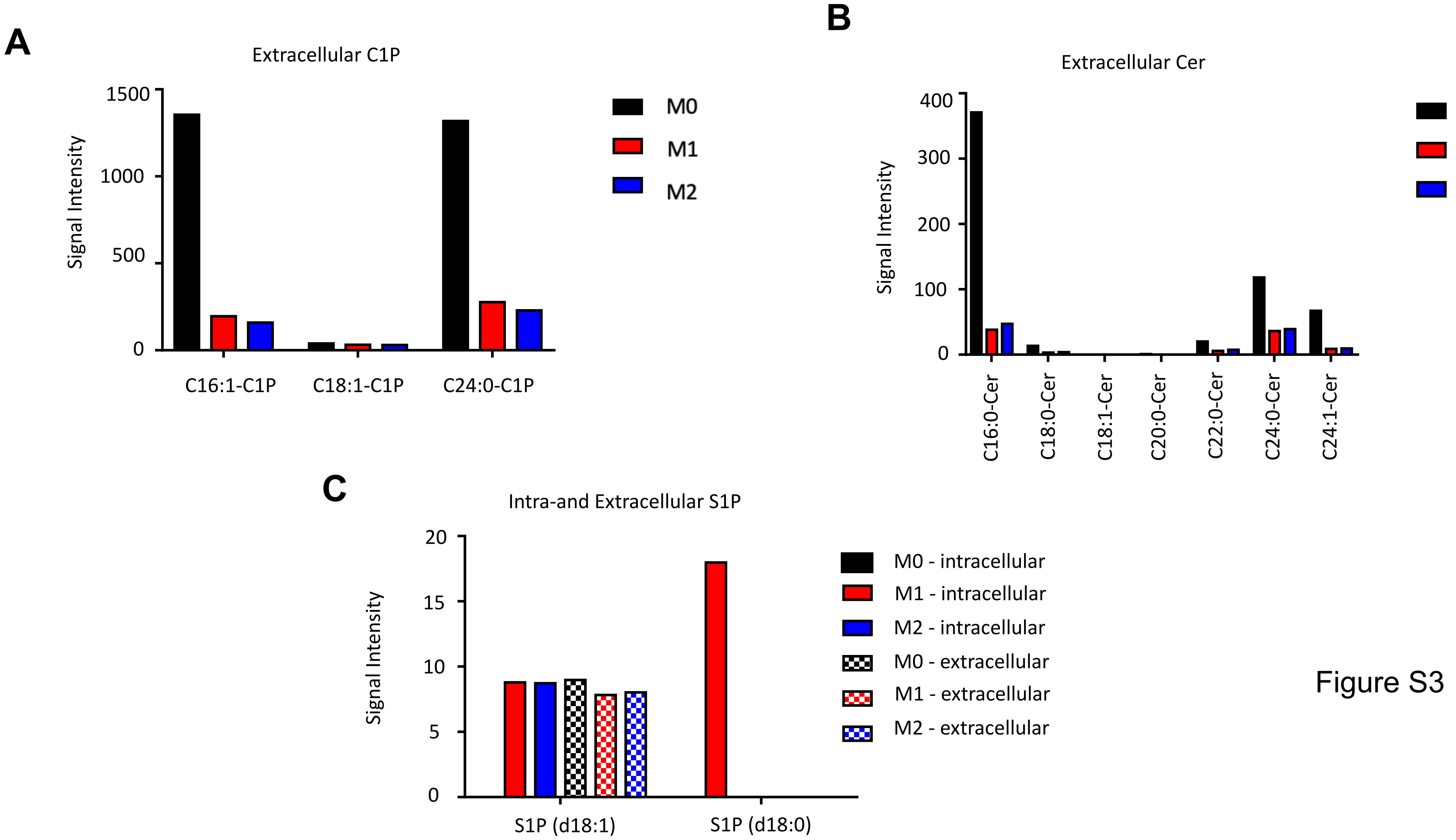
A
Extracellular C1P
Signal Intensity
1500
1000
500
0
M0
M1
M2
C16:1-C1P
C18:1-C1P
C24:0-C1P
B
Extracellular Cer
Signal Intensity
400
300
200
100
0
M0
M1
M2
C16:0-Cer
C18:0-Cer
C18:1-Cer
C20:0-Cer
C22:0-Cer
C24:0-Cer
C24:1-Cer
C
Intra-and Extracellular S1P
Signal Intensity
20
15
10
5
0
S1P (d18:1)
S1P (d18:0)
M0 - intracellular
M1 - intracellular
M2 - intracellular
M0 - extracellular
M1 - extracellular
M2 - extracellular

Figure S3

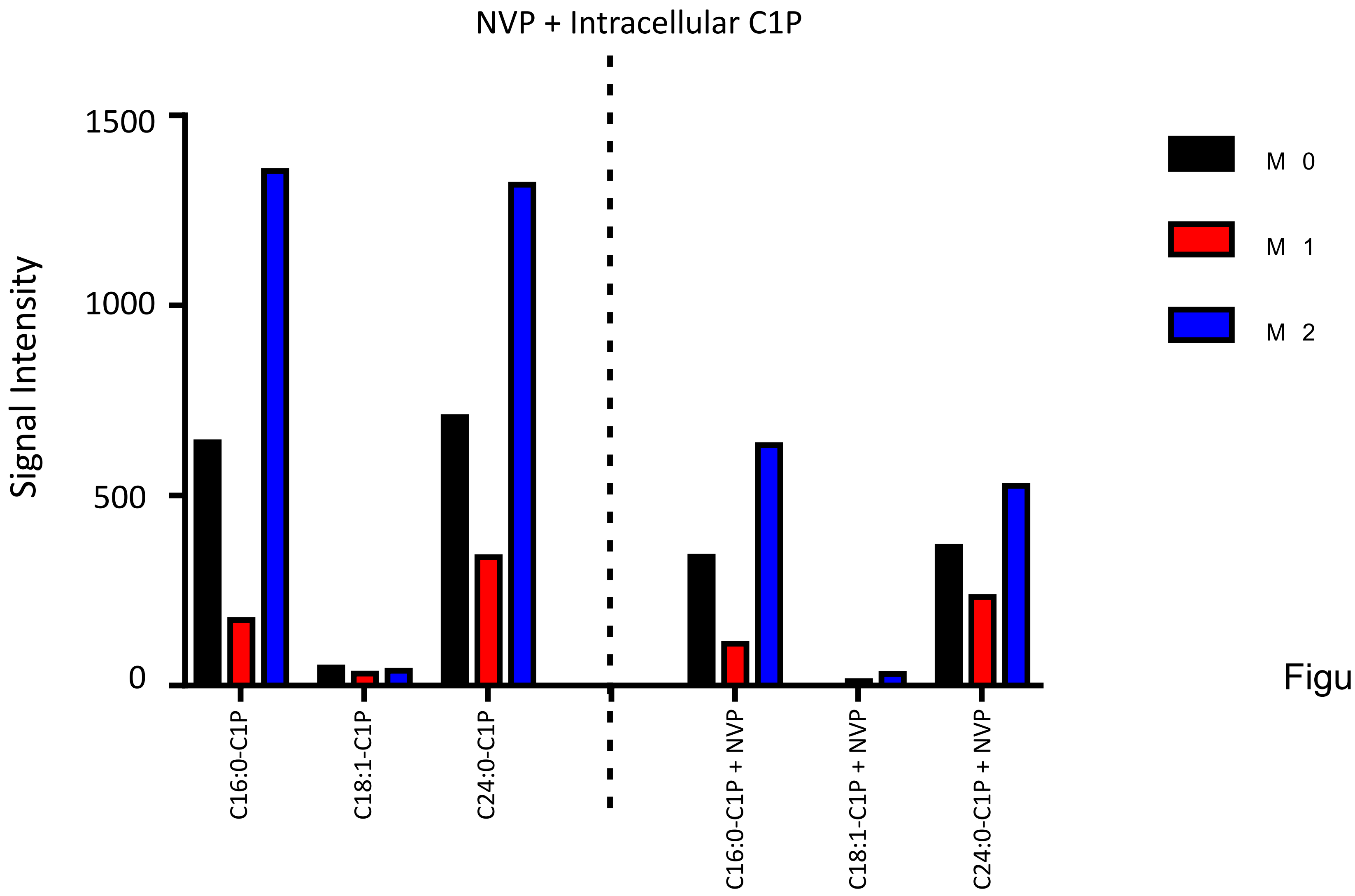

NVP + Intracellular C1P
Signal Intensity
1500
1000
500
0
C16:0-C1P
C18:1-C1P
C24:0-C1P
C16:0-C1P + NVP
C18:1-C1P + NVP
C24:0-C1P + NVP
M 0
M 1
M 2

Figure S4
